# A causal and dissociable role for the right inferior frontal gyrus in empathy for physical and social pain

**DOI:** 10.3758/s13415-025-01388-9

**Published:** 2026-03-20

**Authors:** M. De Lillo, A. Korpal, H. Ferguson, A. K. Martin

**Affiliations:** 1 School of Psychology, University of Birmingham, Birmingham, UK; 2 Cambridge Cognition, Bottisham, UK; 3 School of Psychology, University of Kent, Canterbury, UK; 4 Kent Medway Medical School, Canterbury, UK

**Keywords:** Empathy, Social pain, Physical pain, Dorsomedial prefrontal cortex, Focal transcranial direct current stimulation

## Abstract

The right inferior frontal gyrus (rIFG) and dorsomedial prefrontal cortex (dmPFC) are key nodes in the social brain, implicated in empathy for physical and social pain. However, their causal and dissociable contributions remain unclear. In this study, 52 young adults underwent focal transcranial direct current stimulation (f-tDCS) targeting the rIFG or dmPFC in a sham-controlled, double-blind, crossover design. Participants rated the intensity of pain in images depicting social or physical pain during stimulation. Anodal stimulation of the rIFG increased ratings of physical pain but decreased ratings of social pain, suggesting distinct roles in empathic processing for these two pain types. However, the nonspecific response to physical images indicates that the effect may reflect enhanced attentional capture rather than empathy per se. In contrast, dmPFC stimulation did not modulate ratings, potentially reflecting its role in higher-order social cognitive processes rather than affective empathy. These results provide causal evidence for the rIFG’s role in shaping responses to others’ pain, with effects differing across pain types. While some of these effects may reflect enhanced salience or attentional capture rather than empathy alone, the findings nonetheless support the idea that distinct neural processes contribute to empathy for social versus physical pain.

Empathy refers to the ability to understand and share the mental states of others (Singer et al., 2004) and is the process through which we comprehend both the emotional and physical states of others, including their experience of pain (Decety & Jackson, 2004; Ferguson & Wimmer, 2023; Gallese, 2003; Timmers et al., 2018). Empathy can often be felt for others in physical pain, but we also empathise with people experiencing social pain, such as bereavement or social rejection. Considerable research has explored the shared and unique neural mechanisms underpinning processing of physical and social pain in relation to oneself (Kross et al., 2011; Eisenberger et al., 2012a; Meyer et al., 2015; Ochsner et al., 2008). However, much less is known about the extent to which common and unique neural processes are involved in understanding physical and social pain experienced by others. Causal evidence for the role of key social brain regions in relation to processing physical and social pain in others is lacking.

Although our distinction between empathy for physical and social pain resembles the division between cognitive and affective empathy (Shamay-Tsoory, 2011), the two frameworks address different levels of explanation. The cognitive-affective distinction describes how empathic processing unfolds, whether through embodied emotional sharing or through mental-state inference, regardless of the eliciting stimulus. In contrast, our framework differentiates empathy by domain: the nature of the painful experience being understood (physical versus social). Both physical and social pain can evoke cognitive and affective components, but accumulating evidence suggests that empathy-related responses may also depend on domain-general processes, such as attentional capture, salience detection, or evaluative decision-making (Corbetta & Shulman, 2002; Hampshire et al., 2010; Zaki & Ochsner, 2012). By explicitly distinguishing the domain of empathy from the processes involved, our approach provides a novel theoretical contribution and generates testable predictions about the selective neural mechanisms supporting empathy for social versus physical pain.

In this paper, we address these gaps directly by using focal transcranial direct current stimulation to excite either the right inferior frontal gyrus (rIFG) or the dorsomedial prefrontal cortex (dmPFC) while participants rated the physical or social pain experienced by others. Much of our understanding of how the brain responds to pain in others has been acquired through neuroimaging studies that have focused on empathy for physical pain (Singer et al., 2004; Masten et al., 2011). This work has consistently shown that responding to others in physical pain recruits brain regions such as the anterior insula (AI), the dorsal anterior cingulate cortex (dACC), and neighbouring cortical regions, including the rIFG (Lamm et al., 2011; Masten et al., 2011; Singer et al., 2004). More recent studies have further elaborated the contributions of these regions, including structural and functional associations between the AI and trait empathy for pain (Li et al., 2020), the rIFG’s causal involvement in pain-related affective and perceptual processing (Li et al., 2021), and distributed representations of social pain that extend beyond the classical pain empathy network (Zhang et al., 2025). Notably, the AI and rIFG are anatomically adjacent and functionally interconnected, often co-activating during empathy tasks and forming part of a broader salience and attentional control networks (Menon & Uddin, 2010; Seeley et al., 2007). This raises the possibility that stimulation effects in these regions may reflect enhanced perceptual salience or attentional priority rather than empathy alone.

Research into the affective and evaluative response to social pain in others has received less attention. Social pain has been conceptualised as a painful response to disruptions in one’s social connections, such as social exclusion or rejection (Williams, 2007), or as distress arising when a fundamental need for belonging, control, or meaning goes unmet (Eisenberger, 2012b; Eisenberger, 2015). More recent accounts extend the construct beyond rejection to include a broader class of socially aversive experiences involving social-evaluative threat, such as embarrassment, humiliation, interpersonal conflict, or witnessing one’s own or others’ social mishaps (Krach et al., 2011; 2015; Laneri et al., 2017). Although these phenomena differ in their eliciting contexts, they share a core feature: they signal a disruption or threat to social belonging, esteem, or relational security. In the present study, we use the term *social pain* as an umbrella construct capturing a range of socially negative experiences that evoke distress in response to challenges to one’s social standing or connectedness. We also acknowledge that social pain is heterogeneous and that our stimuli reflect this diversity.

Very little is known about how social pain is processed in the brain. One potential region of interest is the dorsomedial prefrontal cortex (dmPFC), a vital hub in the social brain network (Schurz et al., 2014), implicated in a wide range of higher-order social cognitive processes, including Theory of Mind (Spunt & Adolphs, 2017), perspective-taking (Martin et al., 2017a, 2017b), and self-other processing (Martin et al., 2019a, 2019b; Wittmann et al., 2016). The dmPFC may also play a key role in interpreting the cognitive component of empathy (Masten et al., 2011), particularly when understanding complex, context-dependent forms of social suffering. Notably, Meyer et al. (2013) demonstrated that activity in the dmPFC increases when individuals empathise with the social pain of strangers, suggesting that this region supports the inference of others’ internal states even in the absence of personal familiarity or overt emotional cues. This sensitivity to inferred meaning and context has led to the proposal that dmPFC involvement in social pain may reflect mentalising rather than affective sharing per se. Unlike physical pain, which may be understood through embodied simulation, social pain often requires mentalising and contextual appraisal, functions associated with dmPFC engagement (Lee et al., 2018; Schurz et al., 2014). Therefore, excitatory stimulation of the dmPFC may increase sensitivity to socially relevant information, particularly when processing the social suffering of others.

An unresolved question among empathy researchers is the extent to which neural processes are shared when processing physical and social pain in others (Eisenberg, 2015; Ferguson et al., 2024; Flasbeck & Brüne, 2019; Iannetti et al., 2013). Some studies have shown that overlapping neural areas are recruited in response to others in social and physical pain, despite their diverging phenomenology (Ferguson et al., 2024). Other studies support dissociable roles for these brain regions, with the dorsomedial prefrontal cortex (dmPFC) potentially involved in processing social pain to a greater extent than physical pain (Bruneau et al., 2013; Meyer et al., 2015). The physical-social pain overlap hypothesis (Panksepp, 2003) suggests that social pain relies on some of the same neural regions involved in processing physical pain, particularly the anterior insula (AI) and dACC, as well as neighbouring cortical regions, such as the right inferior frontal gyrus (rIFG) and the dmPFC (Masten et al., 2011). However, other researchers have contested the notion that these systems are completely homogenous (Eisenberger, 2015). Shared neural networks for processing social and physical pain may have evolved as an adaptive mechanism, promoting behaviours that help individuals respond effectively to threats or challenges (Sturgeon & Zautra, 2016). Despite this, most research has focused on self-experienced social and physical pain, with less attention given to how these networks contribute to evaluating others’ pain.

Recent studies have begun addressing this gap by examining empathy for physical and social pain within matched experimental paradigms. Ferguson et al. (2024) used EEG and a pain-rating task with carefully matched stimuli to investigate neural responses associated with empathy for physical and social pain. They found greater mu desynchronization, an EEG marker linked to the human mirror neuron system, in response to pain versus no-pain situations, but no significant differences between physical and social pain. In contrast, Flasbeck & Brüne (2019) identified distinct early (330–450 ms) and late (500–700 ms) event-related potential (ERP) responses, with physically painful stimuli eliciting stronger late ERP responses than socially painful stimuli, particularly in components, such as the late positive potential (LPP). These studies highlight the temporal dynamics of pain-related processes but reveal limitations in determining causal relationships between specific brain regions and these responses. Recent advances in noninvasive brain stimulation have provided causal evidence for the role of the rIFG and related regions in modulating responses to others’ pain. For instance, Wu et al. (2018) showed that excitatory stimulation to the rIFG increased cognitive empathy, while He et al. (2020) demonstrated that stimulating the rVLPFC reduced negative emotional reactions to social pain. However, neither of these studies tested empathy for both physical and social pain within the same task and cohort, and methodological limitations, such as nontargeted stimulation and lack of control sites, limits the interpretability and specificity of the findings.

The present study aims to provide causal evidence for the role of the rIFG and the dmPFC in empathy for social and physical pain in others. These regions were selected as they represent key hubs in the social brain network, with potentially complementary roles in processing the social and physical dimensions of empathy (Bruneau et al., 2013; Lamm et al., 2007; Masten et al., 2011; Meyer et al., 2015; Singer et al., 2004). Furthermore, their anatomical locations are accessible to transcranial direct current stimulation (tDCS), offering promising targets for future neuromodulatory interventions. By employing focal transcranial direct current stimulation (f-tDCS), this study overcomes the limitations of diffuse stimulation methods, such as conventional tDCS, allowing us to isolate the contributions of these specific regions to physical and social empathy.

This investigation directly addresses a key unresolved question in empathy research: to what extent do neural processes overlap when processing physical and social pain in others? We hypothesize that stimulation of the rIFG will have opposite effects on physical and social pain, because this region supports both embodied mirroring of others’ bodily states and top-down regulatory control. Increasing rIFG excitability is therefore expected to amplify responses to physically painful cues. In contrast, the same stimulation is predicted to reduce ratings of others’ social pain, reflecting the rIFG’s established role in inhibitory and regulatory processes that dampen emotional reactions to socially distressing information. Conversely, we predict that stimulation of the dmPFC will preferentially increase empathic responses to social pain, consistent with its involvement in mentalising and socio-evaluative interpretation of complex social interactions, with comparatively smaller or no effects on empathy for physical pain. Finally, by including both painful and nonpainful stimuli, we are able to test whether stimulation effects are specific to empathic processing or whether they generalise to broader changes in salience or evaluative bias.

This study will provide critical insights into the functional specialization and interaction of these regions within the broader empathy network. By testing whether stimulation of these regions produces overlapping or dissociable effects on physical and social pain empathy, the study informs ongoing debates about the shared versus unique neural mechanisms that underlie these processes. If overlapping effects are observed, this would support the physical-social pain overlap hypothesis, suggesting evolutionary and functional commonalities. Alternatively, dissociable effects would indicate distinct neural pathways for processing physical and social pain, reinforcing the idea that these experiences engage unique cognitive and affective mechanisms. By isolating the contributions of these brain areas, this research advances our understanding of empathy’s neural architecture and offers potential avenues for future targeted interventions in clinical contexts involving impaired social or emotional processing.

## Methods

Power calculations were conducted using the *webpower* package (Zhang & Yuan, 2018) in R. Based on prior f-tDCS studies reporting large between-group behavioural effects in social cognition (Martin et al., 2019a), we estimated that 52 participants (26 per stimulation condition) would provide 80% power to detect a large between-subjects effect of stimulation (Cohen’s f = 0.40, α = .05). This calculation was designed to ensure adequate power for the primary between-group comparison. Owing to the repeated-measures structure of the task, the design is also sensitive to moderate within-subject effects involving Pain Type and Content. However, the higher-order interactions examined in the present study (e.g., Stimulation × Type × Content) are expected to be smaller in magnitude and were not specifically powered for. Accordingly, these interaction findings should be interpreted with caution and ideally replicated in larger samples.

Participants were healthy young adults (18–35 years; predominantly University students), assigned to stimulation over either the dorsomedial prefrontal cortex (dmPFC; *n* = 26) or right inferior frontal gyrus (rIFG; *n* = 26) in a sham-controlled, double-blinded, crossover study. A between-subjects approach to stimulation site (dmPFC vs. rIFG) was adopted to avoid carry-over or interference effects that can arise when multiple cortical sites are stimulated within the same individual. Sequential stimulation of different regions can induce residual neurophysiological changes or alter participants’ expectations, complicating interpretation of site-specific effects. Using separate groups for each site therefore provides a cleaner comparison of functional specificity while maintaining the advantages of the within-subject (active vs. sham) crossover for isolating stimulation effects. Stimulation order was balanced, with half receiving active and half receiving sham stimulation first. Groups were comparable in age, gender, autism-quotient scores (Baron-Cohen et al., 2001), and hospital anxiety and depression scores (Zigmond & Snaith, 1983; Table 1). Participants were tDCS-naïve, with no history of neurological or psychiatric conditions, and were compensated for participation. Ethical approval was obtained from the University of Kent's Research Ethics Committee, following the 1991 Declaration of Helsinki [Ethics ID: 202116387864337438]. All participants provided informed written consent before taking part.

**Table 1:** Demographics across stimulation condition groups

	rIFG M (SD)	dmPFC M (SD)	*p*^a^
Age (yr)	22.16 (3.60)	21.42 (3.69)	.50
Gender	20F/6M	19F/7M	.75
Autism quotient	20.85 (8.65)	19.81 (9.94)	.69
HADS-depression	5.58 (3.67)	6.46 (3.85)	.40
HADS-anxiety	7.81 (4.75)	8.00 (4.49)	.88

^a^ Between-group comparisons.

SD = standard deviation.

## Pain rating task

Participants completed a modified version of the pain rating task (Ferguson et al., 2024; Jackson et al., 2005) using PsychoPy (2022.1.3). The task included 80 images (taken directly from Ferguson et al., 2024), with 20 depicting physical pain, 20 no physical pain, 20 social pain, and 20 no social pain (full set: https://osf.io/f9c2r/). Images were classified as social pain if they depicted interpersonal or relational distress (e.g., rejection, humiliation, or exclusion) without physical injury, and as physical pain if they depicted clear nociceptive harm. Images with overlapping features or ambiguous cues were excluded. The no-pain conditions served as perceptual control images matched for scene content and visual complexity but lacking painful events. Details of a pretest study that identified and selected suitable images are provided in the supplementary materials of Ferguson et al. (2024).

In addition, we conducted further validation of the stimuli in an independent sample (*N* = 61), where participants rated arousal, salience, pleasantness, and visual complexity. As expected, pain images received higher arousal and salience ratings than their matched no-pain images, with smaller differences for social than physical stimuli (see Appendix 1 for full validation data). Importantly, these stimulus characteristics cannot account for the stimulation effects observed in the main study. Given that the images were closely matched on visual complexity and showed only the expected valence and arousal differences between pain and no-pain conditions, any modulation by tDCS is unlikely to be driven by low-level perceptual properties or general emotional reactivity to the stimuli.

**Fig. 1:**
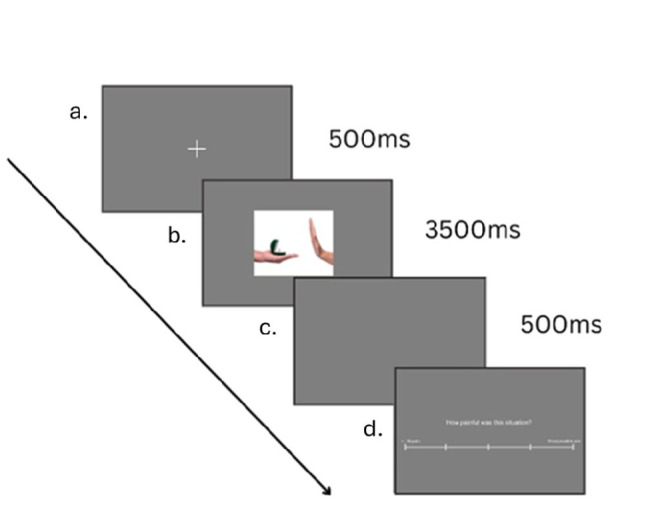
Schematic of the task design and timings. *Note.* **a.** Fixation cross (500 ms); **b.** stimulus (3,500 ms); **c.** blank screen (500 ms); **d.** rating (“How painful was the situation?”)

Participants completed four counterbalanced blocks (320 trials total). Each trial consisted of a fixation cross (500 ms), an image (3500 ms), and a rating screen using a 0–100 visual analogue scale (Fig. 1). Participants were instructed to rate how much pain the person in the image was experiencing, consistent with standard empathy-for-pain paradigms (Singer et al., 2004). Images appeared in random order with a 500-ms intertrial interval, and the task lasted approximately 30 minutes per session.

## Focal-tDCS

Focal-tDCS was delivered by using a DC-Stimulator Plus (NeuroConn) with two concentric rubber electrodes (Bortoletto et al., 2016; Gbadeyan et al., 2016a). The centre electrode (2.5 cm diameter) was placed over the target region, with a surrounding ring electrode (7.5/9.8 cm inner/outer diameter). This setup, akin to the “4 x 1” focal-tDCS montage (Alam et al., 2016; Hogeveen et al., 2016; Kuo et al., 2013), was preferred for cost-efficiency and comparable focal current delivery (Martin et al., 2017b; 2019a; Martin et al., 2019b). Electrodes were secured with conductive gel and an EEG cap, with locations identified using the 10–20 EEG system: dmPFC stimulation was centred 65% along the Cz-Fpz line (Martin et al., 2017a), and rIFG stimulation at FC6 (Hogeveen et al., 2015; 2016). Unlike conventional tDCS, which uses relatively large electrodes that generate a diffuse electric field across broad cortical areas, focal tDCS (f-tDCS) employs smaller high-definition electrodes arranged in a concentric montage to concentrate current over a specific cortical region. This configuration increases the spatial specificity of stimulation, reduces current spread to neighbouring areas, and allows more targeted modulation of neural activity in the intended region. These properties make f-tDCS particularly suitable for causal investigations of region-specific contributions to social-cognitive processes.

Active and sham stimulation was administered across two sessions separated by at least 72 hours. During active stimulation, the current ramped to 1 mA over 5 s, lasting 20 min before ramping down (5 s). Sham stimulation mimicked active stimulation but only lasted 40 s before ramping down. This method effectively blinded participants (Gbadeyan et al., 2016b; Martin et al., 2017a; Martin et al., 2019a; 2019b) with no neurophysiological effect (Stagg et al., 2013). The study was double-blinded using the stimulator’s “study mode,” and impedances were maintained below 55 kΩ for safety. Stimulation order was counterbalanced across sessions and brain regions.

**Fig. 2:**
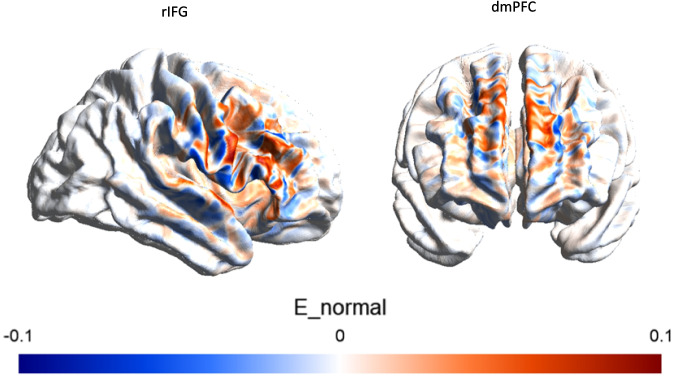
Theoretical distribution of the normal component of the electric field targeting the dorsomedial prefrontal cortex (dmPFC) and right inferior frontal gyrus (rIFG) during online anodal focal transcranial direct current stimulation (tDCS)

SimNIBS (4.1; Thielscher et al., 2015) was used for current modelling, replicating experimental parameters (electrode dimensions, current strength, gel thickness). Standard conductivity values were applied. dmPFC stimulation targeted MNI scalp coordinates (0.5/71.7/46.1), and rIFG stimulation was centered at FC6. Peak E-field magnitudes were 0.13 V/m (rIFG) and 0.09 V/m (dmPFC). We present the normal component of the electric field (Fig. 2), defined as the component perpendicular to the cortical surface, which is thought to more directly influence neuronal excitability and thus better reflect functionally relevant current flow (Radman et al., 2007). Because no MRI data were available to run individualized modelling, the modelling presented in the current study should be considered as approximations of current flow based on a standard brain from a young adult.

## Questionnaires

To ensure rIFG and dmPFC stimulation groups were comparable on autistic traits and mental health characteristics, each participant completed the following questionnaires.

*Autism Spectrum Quotient (AQ).* 50-item self-report scale measuring autism-related traits (range 0–50, higher scores indicate more traits;Baron-Cohen et al., 2001).

*Hospital Anxiety and Depression Scale (HADS).* 14-item scale assessing anxiety (HADS-A) and depression (HADS-D) (range: 0–21 per subscale, higher scores indicate greater severity;Zigmond & Snaith, 1983).

*Mood Change and Adverse Effects.* Mood was assessed pre- and poststimulation by using the Visual Analogue Mood Scale (VAMS; Folstein & Luria, 1973). Participants rated mood descriptors (happy, energetic, afraid, tense, angry, tired, confused, sad) on a visual scale between a neutral and corresponding emotional emoji. Adverse effects were measured post-stimulation (Brunoni et al., 2011) by rating side effects (headache, scalp pain, tingling, etc.) on a 1–4 scale (absent-severe). A total adverse effects score was computed.

## Procedure

Participants attended two lab-based sessions at least three days apart. Session 1 included informed consent, demographics and safety screening, and completion of AQ, HADS, and VAMS. Stimulation was applied during the pain rating task. Post-task, participants completed VAMS again and an adverse effects questionnaire. Session 2 replicated Session 1 assessments (VAMS pre- and post-task, adverse effects questionnaire). After the second session, participants indicated their perceived real stimulation session for blinding assessment. Finally, participants were debriefed and compensated.

## Statistical analysis

Analyses were conducted by using JASP (0.18.3; JASP Team, 2024) and R (4.3.2). A 2x2x2x2 mixed ANOVA examined the effects of stimulation (sham vs. anodal) at different brain regions (rIFG vs. dmPFC) on social and physical pain ratings. Within-subject factors included stimulation (sham vs. anodal), pain type (pain vs. no pain), and content (social vs. physical), with pain ratings (0–100) as the dependent variable.

## Results

Blinding was achieved as participants were unable to guess the real stimulation session better than chance at either the rIFG (50% guessed correctly, χ^2^(1) = 0.00, *p* = 1.0) or the dmPFC (58% guessed correctly, χ^2^(1) = 0.62, *p* = .43).

## Pain ratings

Table 2 presents pain ratings for each condition and stimulation site. A mixed ANOVA was conducted to assess how anodal stimulation to either the dmPFC or rIFG modulated ratings of social or physical pain. A main effect of TYPE demonstrated that participants distinguished painful from non-painful stimuli (pain > no pain), *F*(1, 50) = 1086.24, *p* < .001, ηₚ^2^ = 0.96. A Type × Content interaction, *F*(1, 50) = 45.85, *p* < .001, ηₚ^2^ = 0.48, showed that this pain difference was larger for physical content (*t* = 31.64, *p* < .001, *d* = 4.49) than for social content (*t* = 24.57, *p* < .001, *d* = 3.49). This interaction was further subsumed under a four-way interaction between Brain Region × Stimulation × Type × Content, *F*(1, 50) = 4.34, *p* = .04, ηₚ^2^ = 0.08. To investigate this interaction, separate repeated-measures ANOVAs were conducted for each brain region.

**Table 2:** Mean pain ratings across all conditions and stimulation sites

	rIFG		dmPFC	
	Sham M (SD)	Anodal M (SD)	Sham M (SD)	Anodal M (SD)
Social pain	55.75 (15.96)	50.32 (17.03)	54.40 (12.86)	54.08 (12.25)
No social pain	11.11 (10.69)	10.48 (11.20)	9.63 (9.32)	9.64 (9.21)
Physical pain	60.09 (17.55)	63.56 (18.06)	61.69 (13.83)	61.68 (13.86)
No physical pain	5.08 (4.66)	6.85 (8.73)	5.62 (6.53)	5.77 (7.23)

SD = standard deviation.

**Fig. 3:**
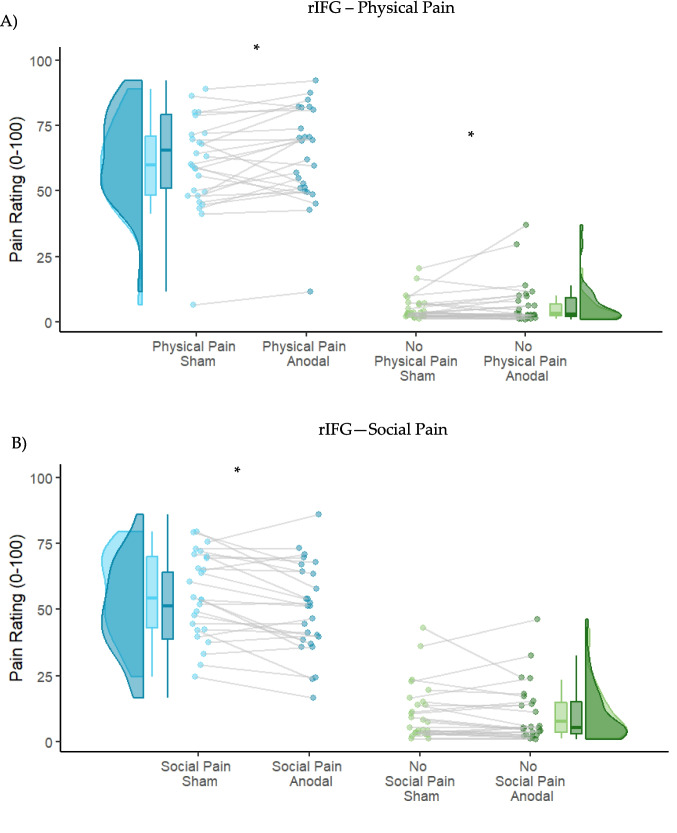
Effects of anodal stimulation to the rIFG on physical and social pain ratings. **A.** Stimulation to the rIFG increased ratings of pain in images depicting physical pain and control images depicting no physical pain. **B.** Anodal stimulation to the rIFG decreased rating for social pain only in images depicting social pain. *Significant differences (*p* < .05)

At the rIFG, evidence supported an interaction between Stimulation × Content × Type, *F*(1, 25) = 6.91, *p* = .02, ηₚ^2^ = 0.22. Separate analyses were conducted for physical and social content to follow-up this effect. For physical content, the interaction between Stimulation and Type was not supported, *F*(1, 25) = 0.60, *p* = .45, ηₚ^2^ = 0.02. However, a main effect of Stimulation was observed, with anodal stimulation increasing pain ratings overall, *F*(1, 25) = 6.91, *p* = .01, ηₚ^2^ = 0.22. For social content, there was a significant Stimulation × Type interaction, *F*(1, 25) = 7.21, *p* = .01, ηₚ^2^ = 0.22. Simple effects analyses showed that anodal stimulation to the rIFG reduced ratings for images depicting social pain, *t* = 3.80, *p* < .001, *d* = 0.39, with no significant effect for social no-pain images, *t* = 0.44, *p* = .67, *d* = 0.05 (Fig. 3).

For the dmPFC, the evidence supported the null hypothesis for all effects of stimulation. Specifically, the interaction between Stimulation x Content x Type was not supported, F(1,51) = 0.01, *p* = .92, η_p_^2^ < 0.001. Likewise, Stimulation x Content, F(1,51) = 0.04, *p* = .84, η_p_^2^ = 0.002, Stimulation x Type, F(1,51) = 0.08*, p* = .78, η_p_^2^ = 0.003, and the main effect of Stimulation, F(1,51) = 0.003, *p* = .96, η_p_^2^ < 0.001, were all nonsignificant. Therefore, stimulation to the rIFG has dissociable effects on images depicting social and physical pain with a reduction in pain rating in images depicting social pain and a general increase in the rating of physical pain regardless of whether the image depicted pain or not. Stimulation to the dmPFC had no effects. 

## Pain ratings over trials

**Fig. 4:**
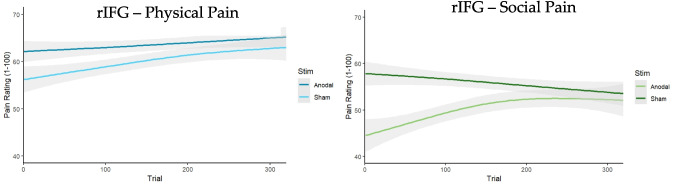
Effect of anodal stimulation to the rIFG on physical (left panel) and social (right panel) pain over trials. *Note.* Anodal stimulation to the right IFG reduced social pain rating disproportionately in the earliest presentations of painful images. In contrast, stimulation consistently increased ratings of physical pain over trials.

To further explore the significant rIFG stimulation effects in opposing directions for social and physical pain, we conducted an exploratory analysis of how these stimulation effects unfolded over trials. This analysis was motivated by evidence that repeated exposure to others’ pain can lead to declining empathic responses over time, a pattern often interpreted as habituation or reduced motivational salience. We therefore tested whether ratings decreased across successive presentations and whether stimulation altered this temporal trajectory. We computed a linear mixed-effects model using the LMER package (Bates et al., 2015) in R. *P*-values were obtained by using the Satterthwaite approximation of degrees of freedom via the lmerTest package (Kuznetsova et al., 2017). The analysis was performed separately for physical and social pain. To examine whether the relationship between Trial and Rating was better characterised by a linear or quadratic function, we compared two mixed-effects models. Both models included Stimulation as a fixed effect and random intercepts and slopes for participants, the first model included a linear fixed effect of trial, whereas the second model additionally included a quadratic term for Trial. Pain ratings over trials are plotted for each stimulation condition and social/physical content in Fig. 4.

For social content, model comparison using a likelihood ratio test revealed that the quadratic model provided a significantly better fit to the data (χ^2^(1) = 6.00, *p* = .014). The Akaike’s Information Criterion (AIC) values also supported the quadratic model (AIC = 38860) over the linear model (AIC = 38856). These results suggest that the relationship between Trial and Rating follows a quadratic trajectory rather than a linear one. The Stimulation x Trial interaction was significant, ß = −3.87, standard error (SE) = 0.79, t(1, 4130.2.1) = −4.90, *p* < .001, showing that stimulation had a disproportionately stronger effect on reducing social pain ratings among earlier trials. There was also a significant effect of Stimulation, ß = 5.44, SE = 0.79, t(1, 4130.1) = 6.89, *p* < .001, indicating that ratings in the sham condition were approximately 5.4 points higher than in the anodal stimulation condition. For physical content, model comparison using a likelihood ratio test revealed that the quadratic model did not provide a significantly better fit to the data (χ^2^(1) = 0.14, *p* = .71). The AIC values also supported the linear model (AIC = 37553) over the quadratic model (AIC = 37555). These results suggest that the relationship between Trial and Rating is best characterized as linear rather than quadratic. The effect of Trial was not significant, ß = −2.23, SE = 1.28, t(1,28.64) = −1.75, *p* = .09. Likewise, the Trial x Stimulation interaction was not significant, ß = 0.72, SE = 0.67, t(1,4160.6) = 1.08, *p* = .28. There was however a significant effect of Stimulation, ß = −3.50, SE = 0.67, t(1, 4108.8) = −5.21, *p* < .001, indicating that ratings in the sham condition were approximately 3.5 points lower than in the anodal stimulation.

## Mood change and adverse effects

As shown in Table 3, Stimulation had no effect on mood change as measured by the VAMS (negative: *F*(1, 50) = 0.39, *p* = .54, ηₚ^2^ = 0.01; positive: *F*(1, 50) = 0.71, *p* = .40, ηₚ^2^ = 0.01) and did not interact with Stimulation site (negative: *F*(1, 50) = 0.73, *p* = .40, ηₚ^2^ = 0.01; positive: *F*(1, 50) = 1.24, *p* = .27, ηₚ^2^ = 0.02). There was also no effect of Stimulation on adverse effects, *F*(1, 50) = 0.05, *p* = .83, ηₚ^2^ < 0.001, and no interaction with Stimulation site, *F*(1, 50) = 0.01, *p* = .91, ηₚ^2^ < 0.001.

**Table 3:** Mood change and Adverse effects across stimulation sites and stimulation types

	rIFG		dmPFC	
	Sham M (sd)	Anodal M (SD)	Sham M (SD)	Anodal M (SD)
VAMS-negative	−1.35 (5.79)	−0.01 (5.68)	−0.49 (3.97)	−0.71 (5.31)
VAMS-positive	−2.02 (3.41)	−2.19 (2.92)	−1.73 (2.14)	−0.47 (2.76)
Adverse effects	4.42 (2.94)	4.54 (3.42)	3.58 (3.37)	3.62 (2.58)

Visual Analogue Mood Scale (VAMS) scores represent a change from pre to post levels of mood.

SD = standard deviation.

## Discussion

Building on research showing overlapping neural processes associated with personal experiences of physical and social pain, it has been claimed that empathy for physical and social pain in others also relies on similar underlying neural processes (Iannetti et al., 2013). However, causal evidence has been lacking. The aim of this study was to provide site-specific causal evidence for the roles of the rIFG and dmPFC in empathic judgments for social and physical pain in others using focal tDCS. Our results support a site-specific, dissociable role for the rIFG, with excitation decreasing ratings of social pain and increasing ratings of physical pain.

These results align with research implicating the rIFG in down-regulating social pain (He et al., 2018; 2020). He and colleagues (2018; 2020) used electrical and magnetic stimulation while presenting images depicting social exclusion, requiring participants to reappraise scenarios with alternative explanations. Excitation of the rIFG improved reappraisal, reinforcing its role in social pain regulation. Previous evidence has found specific evidence for the rIFG in social processing, for example, the rIFG has been linked to social meaning processing (Martin et al., 2016; Tylén et al., 2016) in humans and is active in primates during social but not physical interactions (Sliwa & Freiwald, 2017). Building on this research, the present study is the first to provide causal evidence for a dissociable role of the rIFG in empathy for social and physical pain in the same task. Our findings contribute to this growing body of evidence, underscoring the importance of the rIFG in navigating complex social interactions.

Our findings align with research indicating that processing social pain in others engages neural pathways distinct from those involved in perceiving physical pain, likely reflecting pathways associated with affective processing and regulation. Woo et al. (2014) identified separate neural representations for physical pain and social rejection, with rIFG activity negatively correlated with the dACC, a region implicated in personal social pain. The rIFG may regulate social pain by exerting top-down inhibitory control over the dACC, aligning with its established role in response inhibition (Aron et al., 2014) and emotion regulation (Ochsner et al., 2012). Future research should explore rIFG-dACC connectivity to clarify mechanisms underlying empathy and emotional regulation in social contexts. Understanding these pathways could inform interventions for conditions like social anxiety and depression (Hudd & Moscovitch, 2020; Kupferberg & Hasler, 2023).

The rIFG is also a key hub of the human mirror neuron system (Betti & Aglioti, 2016; Gallese, 2006) and has been linked to processing physical pain in oneself and others (Baird et al., 2011). Observing others in pain increases rIFG activation (Budell et al., 2010, 2015), and disrupting the rIFG via TMS slows pain perception in others (Li et al., 2021). Our study is the first to investigate whether rIFG excitation heightens the perception of physical pain in others. While we found an increase in pain ratings, this effect was not limited to painful images. Even images depicting no physical pain (e.g., near misses with sharp objects) led to increased ratings, suggesting rIFG excitation amplifies threat detection. The rIFG has been linked to threat detection (Clark et al., 2012) and increased attentional sensitivity (Coffman et al., 2012; Falcone et al., 2012). This suggests that excitation enhances perceptual awareness of potential harm, heightening perceived discomfort even in the absence of actual pain.

The dmPFC is associated with higher-order social cognition (Schurz et al., 2014), including empathy (Engen & Singer, 2013), and has been proposed as a target for neurostimulation treatments to enhance empathic function (Phillips et al., 2023). However, our study found no effects of dmPFC stimulation on empathy ratings for social or physical pain. The nature of our task, which focused on affective responses to static decontextualised images, may not have engaged the higher-order cognitive processes underpinning empathy, typically associated with the dmPFC (Martin et al., 2019a; Wittmann et al., 2021). These findings suggest that the efficacy of dmPFC-targeted interventions may be contingent upon task demands that more robustly recruit higher-order cognitive components of empathic processing.

We expected a general habituation to painful images over time, leading to lower pain ratings. However, this pattern was only observed for social pain, with no evidence of change across trials for physical pain in the updated analysis. One possible explanation is that empathic responses to social pain may weaken due to neural habituation, a process in which the brain gradually allocates fewer resources to repeated stimuli (Wilson & Linster, 2008). Such adaptation may be functional in contexts where pain cannot be alleviated (Summerfield et al., 2008). Although neural habituation to pain is well documented (Coll et al., 2016; Preis et al., 2015), behavioural effects on empathy are less consistent (Lamm et al., 2007). Our exploratory findings suggest that habituation during repeated exposure to social pain may differ from responses to physical pain, but further work is needed to establish the reliability and mechanisms of this effect.

If habituation contributes to the reduction in social pain ratings over time, it could also help explain why stimulation effects appeared to diminish across trials. Preis et al. (2015) reported neural habituation in the right insula extending to the rIFG following repeated exposure to painful images, but whether social and physical pain show distinct patterns of habituation at the neural level remains unclear. Given the role of the rIFG in reinterpreting and assigning meaning to social experiences, changes in activation over time could influence the efficacy of stimulation. Because tDCS modulates ongoing neural activity rather than inducing activity directly, reductions in engagement of this region could reduce stimulation effects. Future studies should test whether habituation in rIFG and connected regions contributes to changes in empathy-related behaviour during repeated exposure, ideally using neuroimaging to track neural responses over time.

An alternative interpretation for the reduced stimulation effects in later social pain trials relates to differences between “online” and “offline” stimulation. Online stimulation alters neuronal activity while a cognitive process is underway, whereas offline effects reflect longer-term neuroplastic changes (Liebetanz et al., 2002). In our study, rIFG stimulation may have influenced social pain regulation primarily during online stimulation, with limited carryover effects. Although speculative, this possibility raises important questions about the mechanisms and timing of f-tDCS for social pain and should be examined in future confirmatory studies.

The study should be considered in light of several limitations. Stimulation site was studied as a between-subjects factor and it would increase power if this was within-subjects. The sample was predominantly university students and a more representative sample would benefit future studies. Although our use of real-life photos depicting pain is ecologically valid, future research should enhance ecological validity further. Dynamic stimuli or complex social interactions could assess whether lab-based findings generalize to real-life empathic responses. Social neuroscience is moving toward a ‘second-person’ neuroscience framework (Redcay & Schilbach, 2019), which examines real-time interactions at behavioural and neural levels. Neural synchronization has been identified as crucial for social cognition and interaction (Müller et al., 2021), and the rIFG has been shown to exhibit high levels of interpersonal synchrony (Jasmin et al., 2016; Shamay-Tsoory et al., 2024), suggesting a role in social connectedness. Future studies should employ “second-person” neuroscientific methods to further explore the role of the rIFG in empathy for social and physical pain.

## Conclusion

This study establishes a site-specific, causal role of the rIFG in empathy for social and physical pain. Excitation of the rIFG reduces ratings of social pain while increasing physical pain ratings, even in the absence of actual harm. In contrast, no causal role was found for the dmPFC. Exploratory analyses revealed a stronger stimulation effect on social pain ratings early in the task. These findings refine our understanding of key social brain regions in empathy and challenge shared neural process models for social and physical pain.

## Data Availability

All data and materials are publicly available at OSF (https://osf.io/86sxf/).

## Code availability

All analysis code are publicly available at OSF (https://osf.io/86sxf/).
